# EXPERIENCE OF STROKE RECOVERY FOR WOMEN 60 OR OLDER: USING AUTO-PHOTOGRAPHY TO ENHANCE THE NARRATIVE PROCESS

**DOI:** 10.1093/geroni/igz038.965

**Published:** 2019-11-08

**Authors:** Iona Johnson

**Affiliations:** Towson University, Towson, Maryland, United States

## Abstract

Stroke is a common health concern in the U.S. with 795,000 new strokes each year. Women dominate these numbers, with 55,000 more strokes per year than men, yet they are underrepresented in stroke research. Some research indicates that women have worse physiological and psychosocial outcomes after stroke than men, yet little is known about how they experience recovery. This study used a qualitative phenomenological approach to address the question: “What is the experience of stroke recovery for community dwelling women age 60 or older?” The participants were 10 women, ages 60 – 78, with times post-stroke ranging from 4 months to 15 years. They participated in 2 semi-structured interviews, with auto-photography used to enhance sharing of information. Between the two interviews, they were provided with a digital camera and asked to take pictures that helped to explain their lives before and after stroke. During the 2nd interview, participants described their pictures, and answered additional questions about their recovery. Interviews were transcribed verbatim, and the narratives were coded and analyzed thematically to describe how this sample of individuals experienced stroke recovery. Four overarching themes emerged from the data: 1) the stroke event, 2) a new chapter, 3) meaning and process of recovery, and 4) self-identity. In general, narratives revealed that recovery is described as a complex, individualized, and subjective experience that extends beyond overt physical abilities. Participants in this study experienced changes in self-identity and described a “new normal” after stroke. Implications and recommendations for rehabilitation, research, and policy are discussed.

